# Supplementary material for: Potent antitumour activity of interleukin-2-Fc fusion proteins requires Fc-mediated depletion of regulatory T-cells
Source: Nat Commun. 2017 May 12;8:15373. doi: 10.1038/ncomms15373 (PMC5437307; doi:10.1038/ncomms15373)
Supplement: Supplementary Information — Supplementary Figures, Supplementary Discussion and Supplementary References [file ncomms15373-s1.pdf]

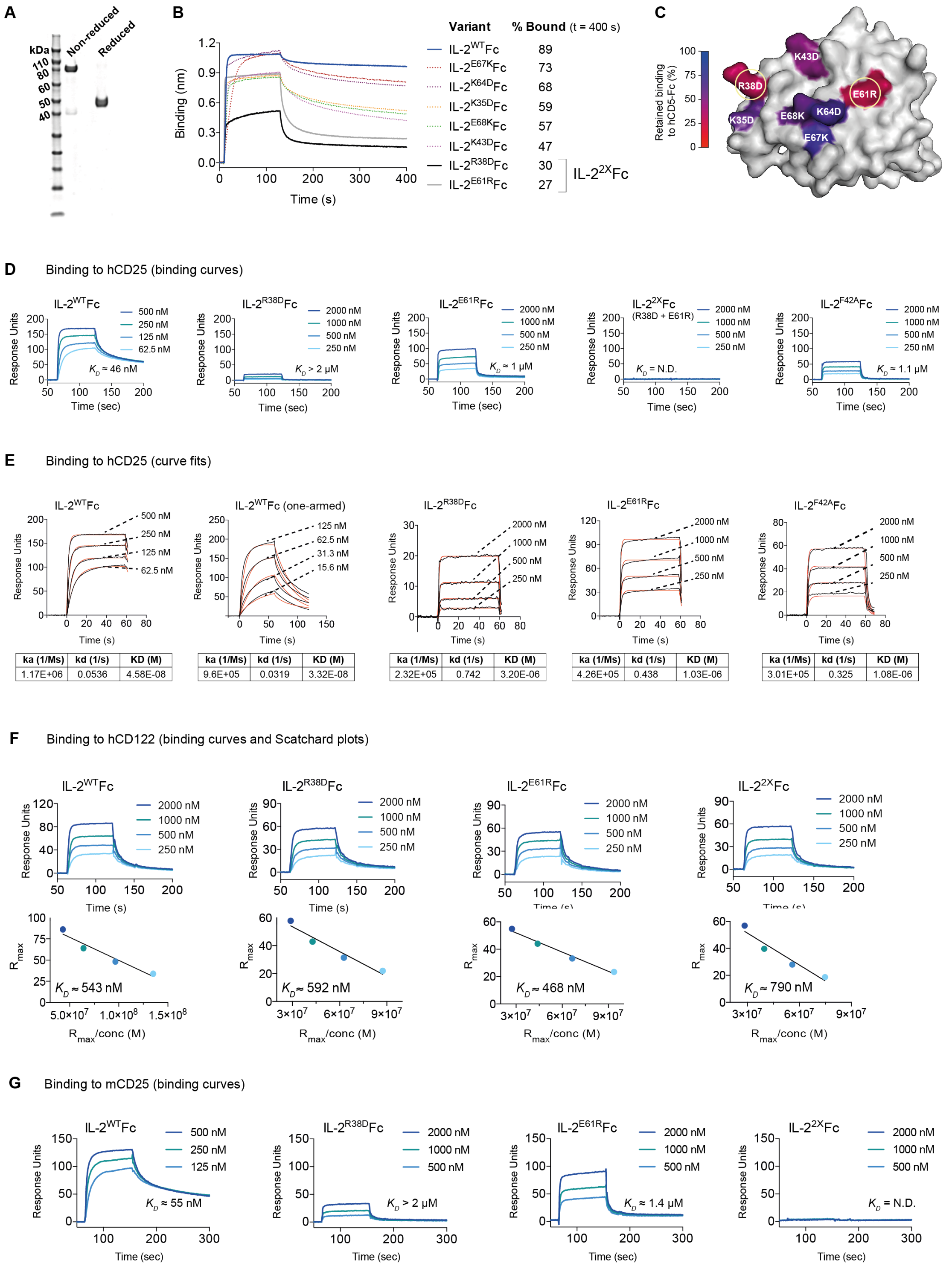

Supplementary Figure 1: Effect of charge-reversal mutations on CD25 and CD122 binding. (A) SDS-PAGE of purified recombinant IL-2-Fc under non-reducing or reducing (10 mM DTT) conditions. (B) Biolayer interferometry was used for ranking of IL-2-Fc proteins containing single charge-reversal mutations in terms

of binding to a bivalent human CD25 Fc fusion protein. Relative binding was estimated by dividing the signal amplitude after dissociation in buffer (t = 400 s) over the maximum signal observed during association (R_{max}). (C) Heat map representation of BLI data showing the location of mutated residues on the hIL-2 surface. IL-2^{R38D}Fc and IL-2^{E61R}Fc displayed the lowest levels of retained binding to hCD25-Fc and were combined to generate a double mutant, IL-2^{2X}Fc. (D) IL-2^{WT}Fc, IL-2^{R38D}Fc, IL-2^{E61R}Fc, IL-2^{2X}Fc and a Fc-fused version of the previously reported[1] IL-2^{F42A} were assessed for binding to monovalent human CD25 using surface plasmon resonance (SPR). (E) The affinity of IL-2^{WT}Fc (bivalent and one-armed), IL-2^{R38D}Fc, IL-2^{E61R}Fc and IL-2^{F42A}Fc to hCD25 was calculated using the 1:1 Langmuir binding model (fits shown in red). For bivalent IL-2-Fc, the binding model was applied to the association period plus the initial two seconds of dissociation. Highly similar K_D values were obtained for IL-2^{WT}Fc (K_D = 46 nM) and one-armed IL-2^{WT}Fc (K_D = 33 nM). (F) SPR analysis of IL-2-Fc binding to immobilized hCD122 (top row). Relative K_D values were approximated by Scatchard analyses of R_{max} vs. R_{max}/concentration plots (bottom row). (G) Binding of IL-2-Fc variants to immobilised mouse CD25 (mCD25), as determined by SPR. K_D values were calculated as described in (E).

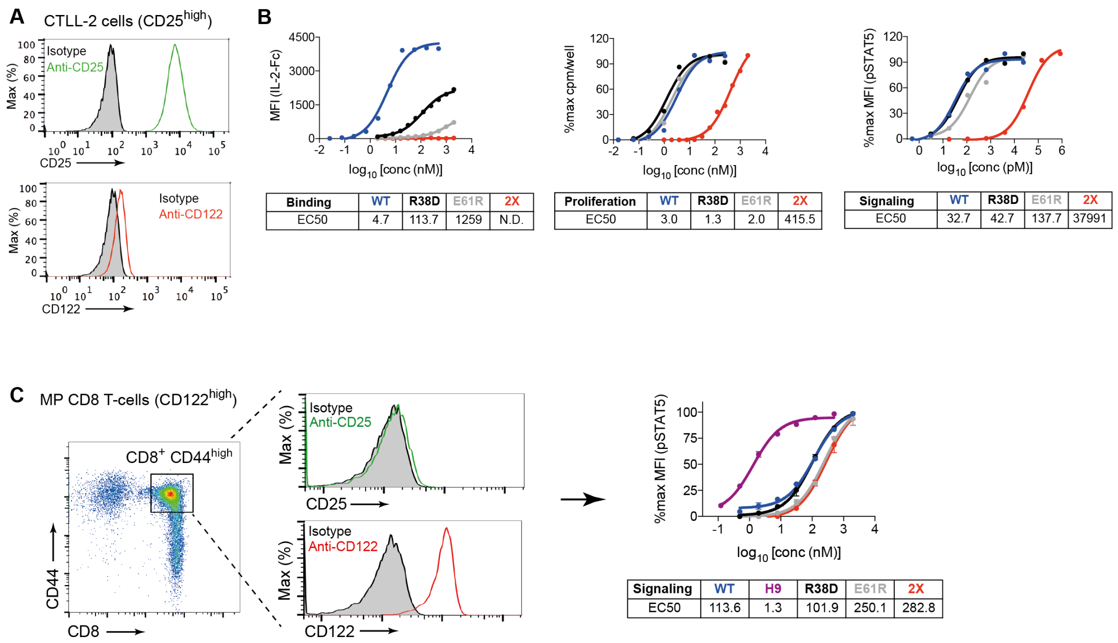

Supplementary Figure 2: *In vitro* activity of IL-2-Fc variants on CD25^{high} and CD122^{high} cells. (A-B) Activity of IL-2-Fc fusion proteins on the CD25^{high} murine CTLL-2 cell line. (A) Histograms showing the levels of CD25 and CD122 on the surface of CTLL-2 cells, as determined by flow cytometry. (B) Left: levels of surface-bound IL-2-Fc after incubation with CTLL-2 cells, as detected by flow cytometry using a fluorescently labelled anti-mIgG2a antibody. Middle: cell proliferation induced by IL-2-Fc variants, as measured by ^{3}H thymidine incorporation. Right: flow cytometric detection of intracellular pSTAT5 in response to IL-2-Fc stimulation. Non-linear least squares fits and EC50 values were computed using the Prism software (GraphPad); data are representative of at least two independent experiments. (C) Activity of IL-2-Fc variants on CD122^{high} CD25^{-} cells. The IL-2^{H9}Fc variant (engineered for high affinity to CD122) was produced and included as a positive control[2]. Left: pseudocolor dot plot of CD8^{+} enriched splenocytes isolated from an IL-7 transgenic mouse (lymphocyte gate is shown). Histograms show the levels of CD122 and CD25 expression in the CD8^{+} CD44^{high} subset. Right: flow cytometric detection of phosphorylated STAT5 in the CD8^{+} CD44^{high} subset after *ex vivo*

stimulation with IL-2-Fc variants. Displayed are means ± SEM of duplicates, non-linear least squares fits and EC50 values. Data is representative of two independent experiments.

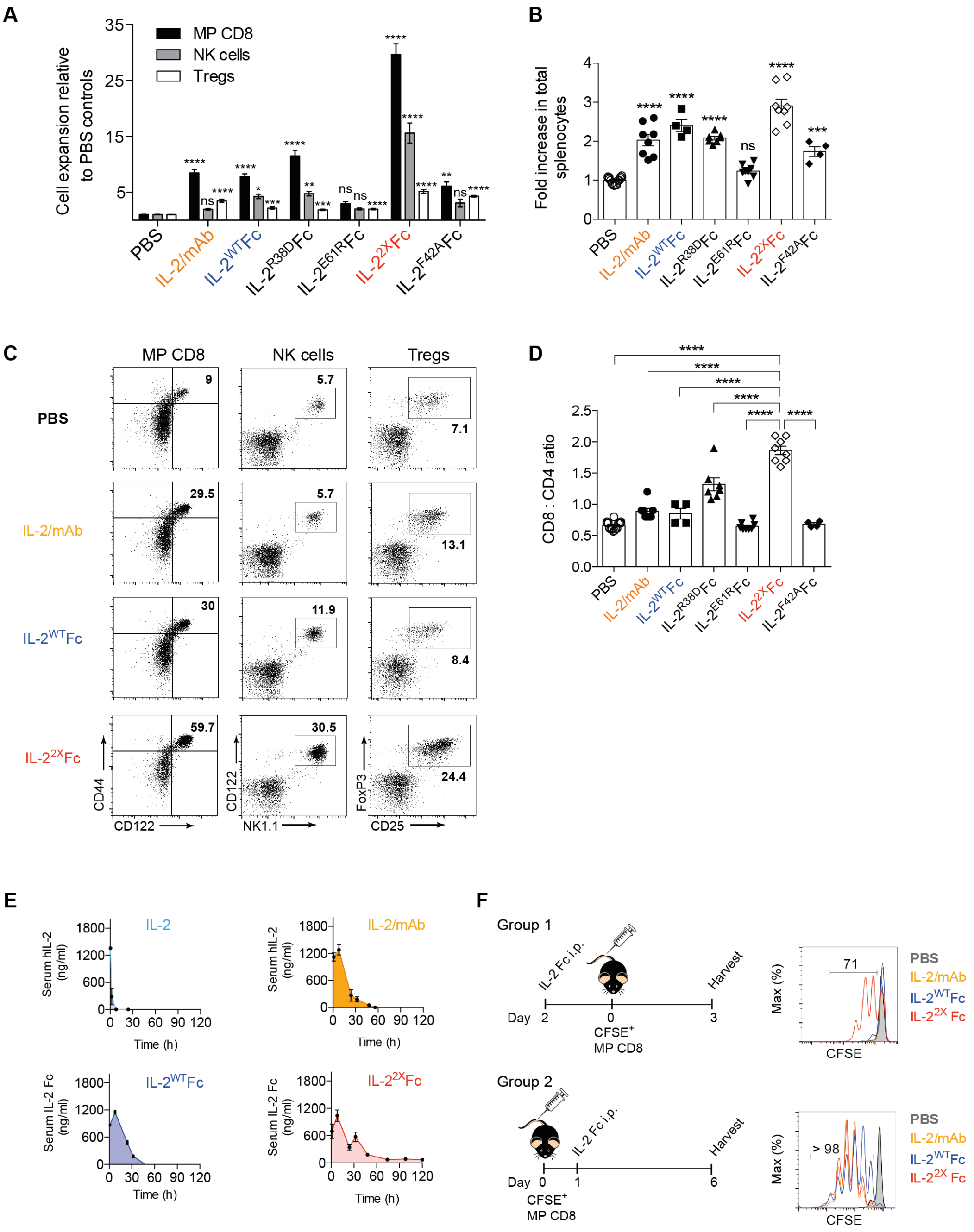

Supplementary Figure 3: IL-2-Fc fusion proteins with increased *in vivo* activity and selectivity. (A-D) Single dose experiments were performed to determine the activity IL-2/mAb and IL-2-Fc variants in C57BL/6 mice. Mice were given IL-2/mAb (3 µg IL-2 + 15 µg mAb), IL-2-Fc molar equivalent (16.8 µg) or PBS control injected i.p. on day 0, followed by harvesting of spleens for flow cytometric analysis on day 5. (A) Fold expansion in the numbers of MP CD8, NK cells and Tregs relative to PBS controls. (B) Fold expansion in the number of total splenocytes relative to PBS

controls. Shown is pooled data from seven independent experiments, each normalized to the average cell numbers of 2-3 PBS-treated mice (PBS: n = 16; treatment groups: n = 4-8). Asterisks indicate significant differences relative to PBS controls. (C) Representative dot plots showing the percentages of MP CD8 ($CD8^+$ gate: $CD44^{high}$ $CD122^{high}$), NK cells ($CD3^-$ gate: $NK1.1^+$ $CD122^{high}$) and Tregs ($CD4^+$ gate: $CD25^+$ $FoxP3^+$) in the spleens of treated mice. (D) $CD8^+$ to $CD4^+$ T-cell ratios in the spleens of treated mice. Shown is pooled data from seven independent experiments (PBS: n = 16; treatment groups: n = 4-8). (E) The serum levels of human IL-2 (20 µg), IL-2/mAb (3 µg IL-2 + 15 µg mAb) and IL-2-Fc variants (16.8 µg) were determined by ELISA at different time points post i.p. injection (n = 2-4). (D) Proliferation of adoptively transferred, CFSE labelled MP CD8 cells in response to IL-2/mAb, IL-2-Fc treatment. C57BL/6 mice received a single dose of IL-2/mAb (3 µg IL-2 + 15 µg mAb) or IL-2-Fc variants (16.8 µg) 48 h prior to MP CD8 transfer (Group 1) or 24 hours post MP CD8 transfer (Group 2). Spleens were harvested 5 days after cytokine treatment for assessment of CFSE dilution in donor MP CD8 cells. Representative histograms are shown (n = 2). Data are displayed as mean ± SEM. Significant differences as determined by one-way ANOVA with Bonferroni post hoc test for multiple comparisons (* $P < 0.05$, ** $P < 0.01$, *** $P < 0.001$, **** $P < 0.0001$).

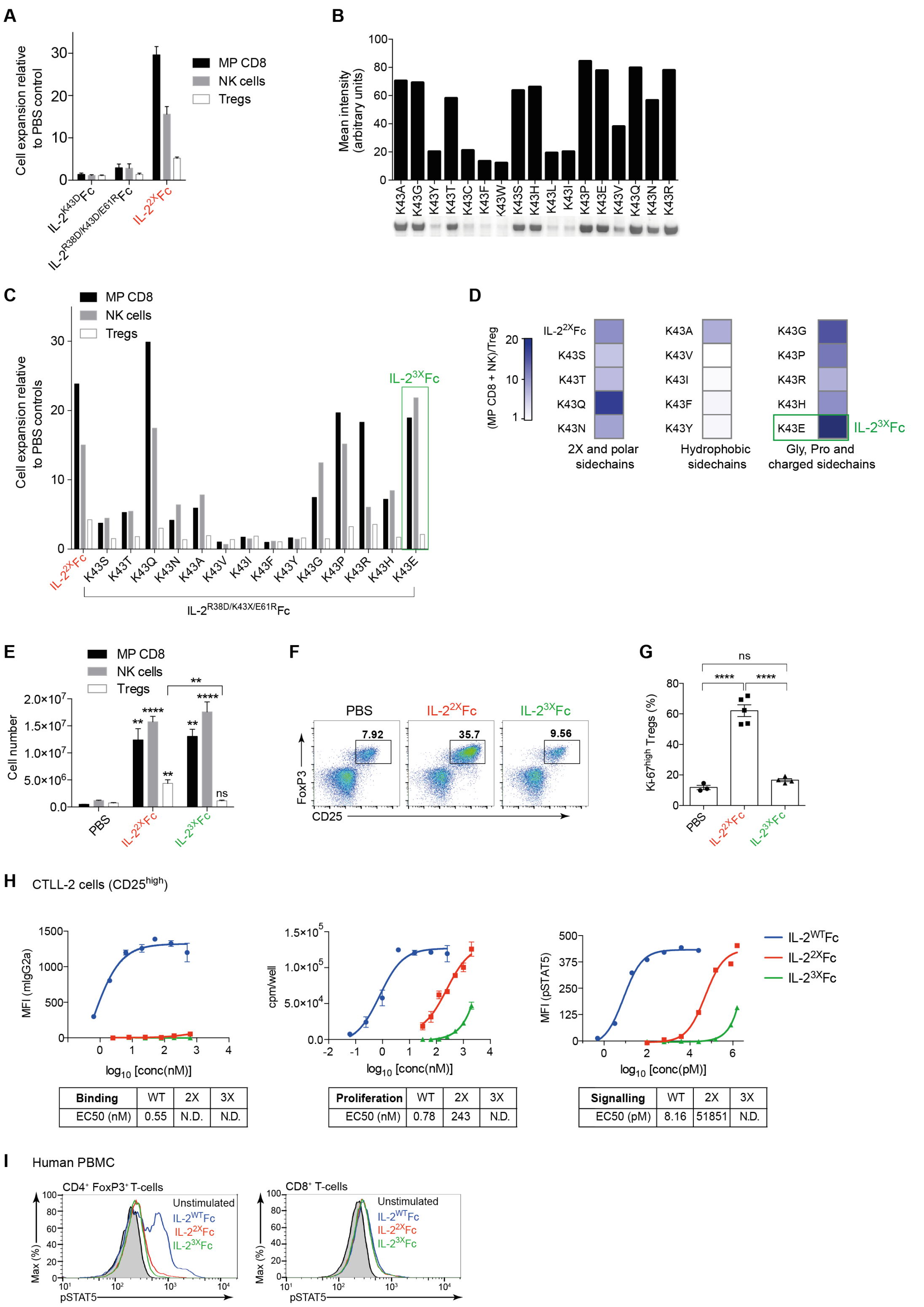

Supplementary Figure 4: Generation and characterization an IL-2-Fc triple mutant with exquisite selectivity for CD122^{high} cytotoxic subsets. (A) Fold expansion in the numbers of MP CD8, NK cells and Tregs in the spleens of C57BL/6 mice treated with a single 16.8 μg dose (i.p., day 0) of IL-2^{K43D}Fc, IL-

$2^{R38D/K43D/E61R}$Fc or IL-2^{2X}Fc were determined by flow cytometry (day 5). Shown is pooled data from three independent experiments, each normalized to the average cell numbers of 2-3 PBS controls (n = 4-8). (B-D) Production and *in vivo* screening of IL-2-Fc triple mutants containing R38D, E61R and K43X mutations (X = any amino acid). (B) SDS-PAGE of produced IL-2-Fc triple mutants. Relative protein expression levels were estimated from densitometry analysis of a Coomassie-stained gel. (C) Fold increase in the numbers of MP CD8, NK cells and Tregs in the spleens of mice treated with a single 16.8 μg dose (i.v., day 0) of IL-2^{2X}Fc or a panel of fourteen IL-2-Fc triple mutants, as determined by flow cytometry (day 5, n = 1). Fold expansion relative to the subset numbers found in a PBS-treated control is shown. Highlighted is the selected IL-2^{3X}Fc containing the mutations R38D, E61R and K43E. (D) Heat map representation of (MP CD8 + NK cell)/Treg ratios calculated from C. The largest ratio was observed for IL-2^{3X}Fc, providing the rationale for its selection (ratio = 19.1). (E-G) Flow cytometric analysis (day 5) of splenocytes harvested from mice treated with PBS or a single 16.8 μg i.p. dose (day 0) of IL-2^{2X}Fc or IL-2^{3X}Fc (n = 3-5). (E) Numbers of MP CD8, NK cells and Tregs in treated mice. Asterisks above bars denote significant differences relative to PBS controls. Asterisks above brackets indicate significant differences between specified groups. (F) Representative pseudocolor dotplots showing the proportion of $FoxP3^+$ $CD25^+$ Tregs in the $CD4^+$ gate. (G) Percentages of highly proliferative Tregs (Ki-67^{high}) in treated mice. (H) Activity of IL-2^{WT}Fc, IL-2^{2X}Fc and IL-2^{3X}Fc fusion proteins in the $CD25^{high}$ CTLL-2 murine cell line. Binding (left), proliferation (middle) and signalling (right) assays were performed as described in Figure S2A. (I) Flow cytometric analysis of STAT5 phosphorylation in human $CD4^+$ $FoxP3^+$ and $CD8^+$ subsets after *ex vivo* stimulation with IL-2^{WT}Fc, IL-2^{2X}Fc or IL-2^{3X}Fc fusion proteins. Data are displayed as mean ±

SEM. Significant differences as determined by one-way ANOVA with Bonferroni post hoc test for multiple comparisons (* $P < 0.05$, ** $P < 0.01$, *** $P < 0.001$, **** $P < 0.0001$).

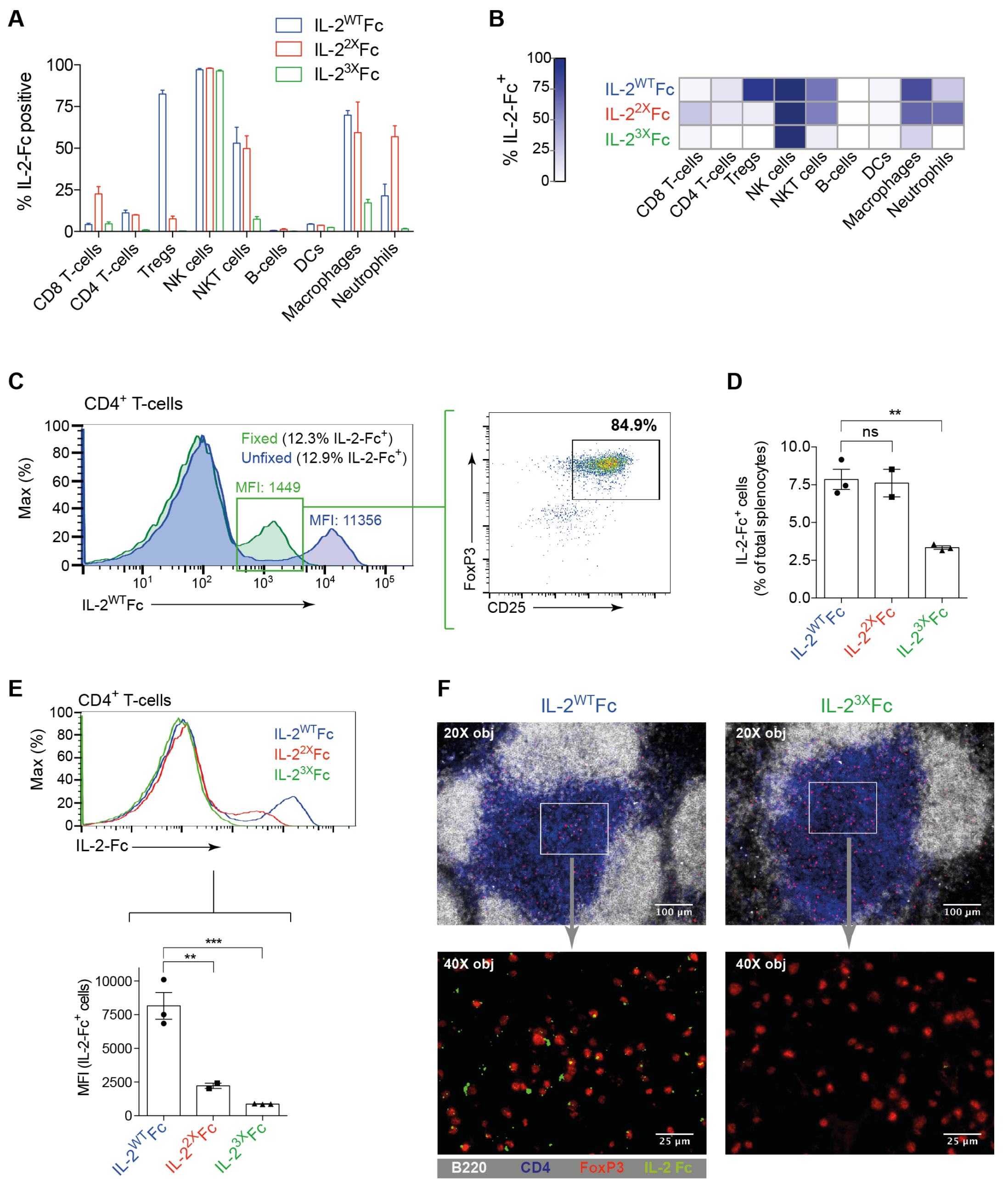

Supplementary Figure 5: IL-2^{3X}Fc displays abolished interaction with regulatory T-cells *in vivo*. (A-D) The cellular biodistribution of fluorescently labelled IL-2^{WT}Fc, IL-2^{2X}Fc and IL-2^{3X}Fc fusion proteins was assessed by flow cytometric analyses of spleens harvested 12 h following injection (16.8 µg, i.p.) of C57BL/6 mice (n = 2-3). (A) Percentages of lymphoid and myeloid subsets bound by labelled IL-2-Fc (B) Heat map representation of the average percentages of IL-2-Fc^{+} cells across different immune subsets. (C) Left: representative histograms comparing the IL-2^{WT}Fc MFI

levels in $CD4^+$ T-cells from unfixed or formaldehyde-fixed (required for FoxP3 intracellular staining) splenocytes. Right: pseudocolor dot plot showing that the majority of $CD4^+$ T-cells bound by IL-2^{WT}Fc belong to the Treg subset ($CD25^+$ $FoxP3^+$). (D) IL-2-Fc^+ cells shown as the percentage of total splenocytes. (E) Top: representative histograms showing the IL-2^{WT}Fc, IL-2^{2X}Fc and IL-2^{3X}Fc MFI levels in the $CD4^+$ T-cell subset. Bottom: quantification of IL-2-Fc MFI in IL-2-Fc^+ $CD4^+$ cells. (F) Fluorescent immunohistochemistry of spleen sections from mice injected with fluorescently labelled IL-2^{WT}Fc or IL-2^{3X}Fc, displaying $CD4^+$, $B220^+$, $Foxp3^+$ and IL-2-Fc^+ cells. Highlighted box (white rectangle) corresponds to a 177 μm x 132 μm area. Data are displayed as mean ± SEM. Significant differences as determined by one-way ANOVA with Bonferroni post hoc test for multiple comparisons (* $P < 0.05$, ** $P < 0.01$, *** $P < 0.001$, **** $P < 0.0001$).

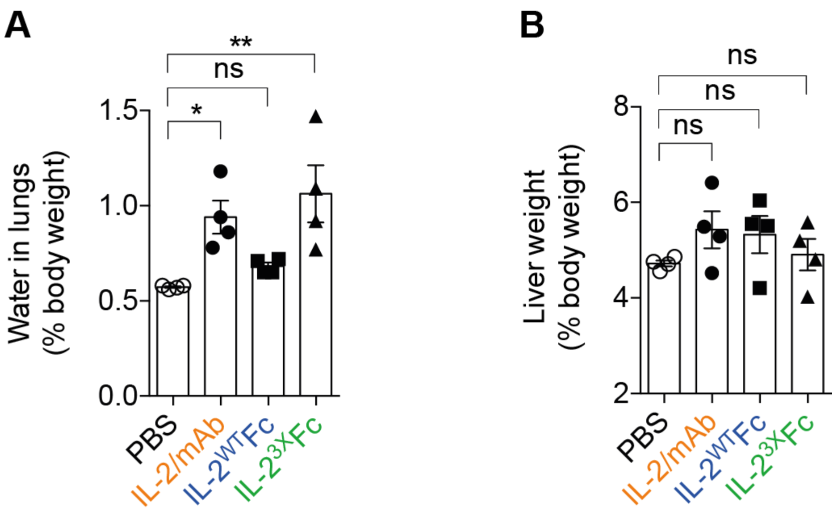

Supplementary Figure 6: Assessment of IL-2/mAb, IL-2^{WT}Fc and IL-2^{3X}Fc treatment toxicity. Evaluation of VLS symptoms in mice receiving five consecutive doses (days 0-4) of IL-2/mAb (1 µg IL-2 + 5 µg mAb per dose), IL-2^{WT}Fc (5.6 µg per dose), IL-2^{3X}Fc (5.6 µg per dose) or PBS control (see Figure 2). (A) Lungs were harvested on day 6 and dehydrated overnight in order to determine water content, shown as the percentage of total body weight. (B) Liver weight (day 6) shown as the percentage of total body weight. Data are displayed as mean ± SEM. Significant differences as determined by one-way ANOVA with Bonferroni post hoc test for multiple comparisons (* $P < 0.05$, ** $P < 0.01$, *** $P < 0.001$, **** $P < 0.0001$).

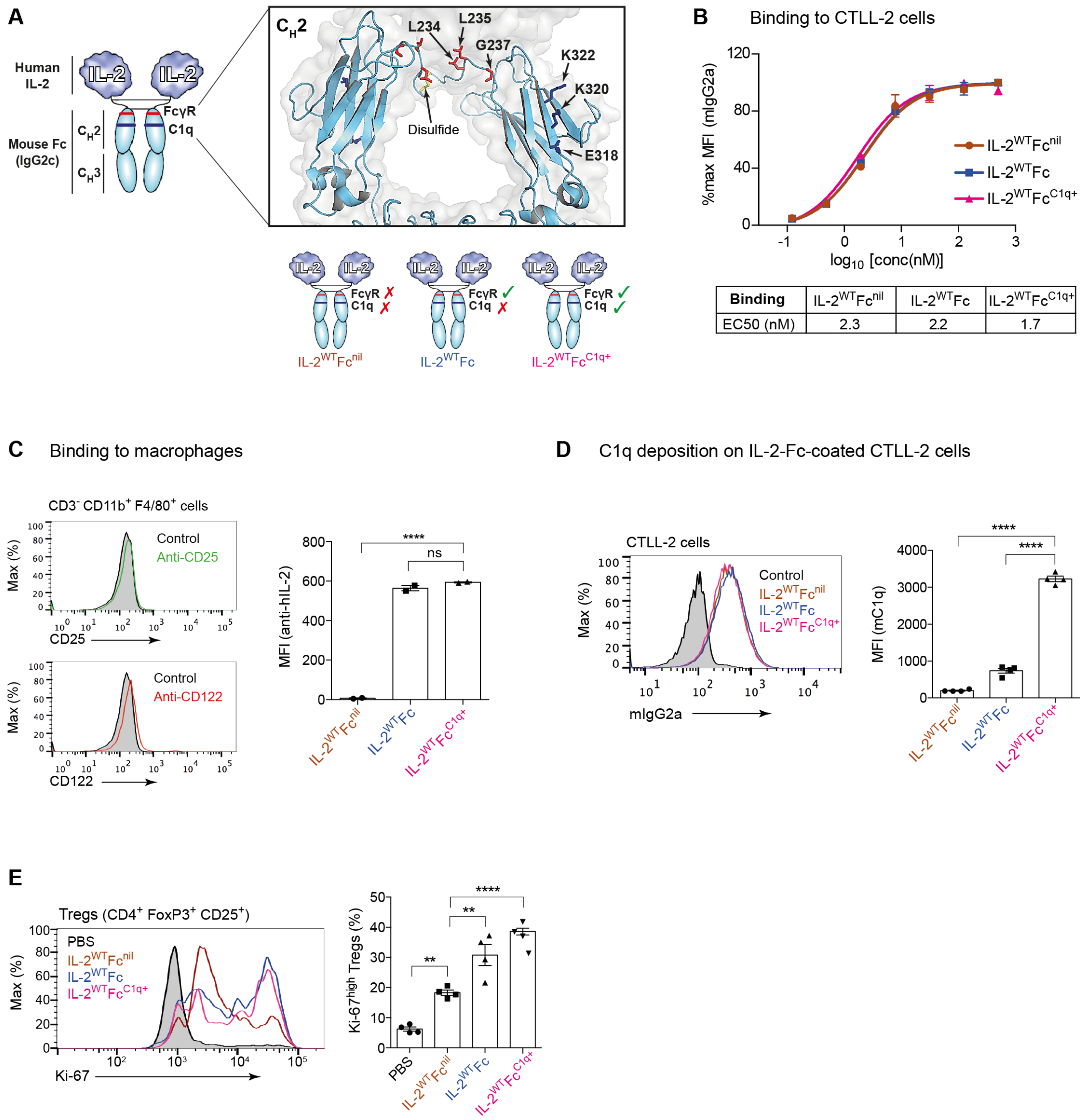

Binding	IL-2WTFcnil	IL-2WTFc	IL-2WTFcC1q+
EC50 (nM)	2.3	2.2	1.7

Supplementary Figure 7: *In vitro* validation and *in vivo* activity of IL-2-Fc proteins with modulated FcγR and C1q binding. (A) Top: the contribution of Fc-mediated effector functions to IL-2-Fc biological activity was studied by introducing previously reported mutations at the FcγR (red) and C1q (blue) binding sites[3,4]. Highlighted residues correspond to Eu numbering[5] and are displayed on a reference crystal structure (human IgG1, PDB: 1E4K[6]). Bottom: Diagram of produced IL-2-Fc variants showing FcγR and C1q binding site status. (B-D) *In vitro* validation of FcγR- and C1q-disrupting mutations within the Fc region of IL-2-Fc. (B) Binding to the CD25^{high} CTLL-2 cell line was performed as previously described. IL-2^{WT}Fc^{nil}, IL-

$2^{WT}Fc$, and IL-$2^{WT}Fc^{C1q+}$ bound CTLL-2 cells with nearly identical EC50 values, indicating that their IL-2 components were unaffected by mutation of the Fc region. Displayed are means ± SEM of duplicates, non-linear least squares fits and EC50 values (n = 2). (C) Binding of IL-2-Fc constructs to C57BL/6 macrophages *ex vivo*, as determined by flow cytometry. Left: representative histograms showing that $CD3^-$ $CD11b^+$ $F4/80^+$ macrophages express negligible levels of IL-2R subunits, thus allowing to attribute IL-2-Fc binding to FcγR present on the surface of these cells. Right: IL-$2^{WT}Fc^{nil}$ was the only construct with a mutated FcγR binding site, resulting in abolished binding to macrophages. (C) Deposition of mouse C1q onto IL-2-Fc-coated CTLL-2 cells, as determined by flow cytometry. Left: All IL-2-Fc constructs coated CTLL-2 cells at equivalent densities. Right: IL-$2^{WT}Fc^{C1q+}$, the only construct with an intact C1q binding site, allowed for high levels of C1q deposition. C1q deposition is substantially reduced on cells coated with IL-$2^{WT}Fc$ and nearly completely abolished on cells coated with IL-$2^{WT}Fc^{nil}$. (E) Flow cytometric analyses of splenocytes (day 5) harvested from C57BL/6 mice treated with five consecutive 5.6 μg i.p. injections of IL-$2^{WT}Fc^{nil}$, IL-$2^{WT}Fc$, IL-$2^{WT}Fc^{C1q+}$ or PBS control on days 0-4 (n = 4, see Figure 3). Left: representative histograms displaying the levels of the proliferation factor Ki-67 in the Treg subset. Right: increased proportions of Ki-67^{high} Tregs after treatment with IL-2-Fc fusion proteins. Data are displayed as mean ± SEM. Significant differences as determined by one-way ANOVA with Bonferroni post hoc test for multiple comparisons (* $P < 0.05$, ** $P < 0.01$, *** $P < 0.001$, **** $P < 0.0001$).

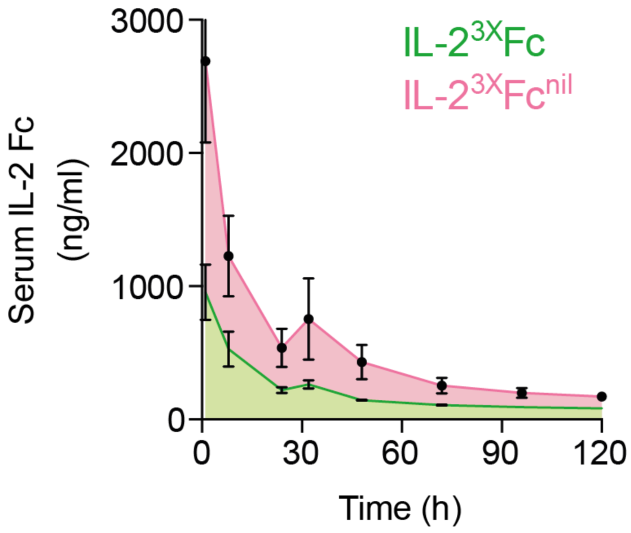

Supplementary Figure 8: IL-2^{3X}Fc^{nil} is detected at higher levels in serum than IL-2^{3X}Fc. The serum levels of IL-2^{3X}Fc and IL-2^{3X}Fc^{nil} were determined by ELISA at different time points after a single 16.8 µg i.p. injection (n = 5 mice per group). Data are displayed as mean ± SEM.

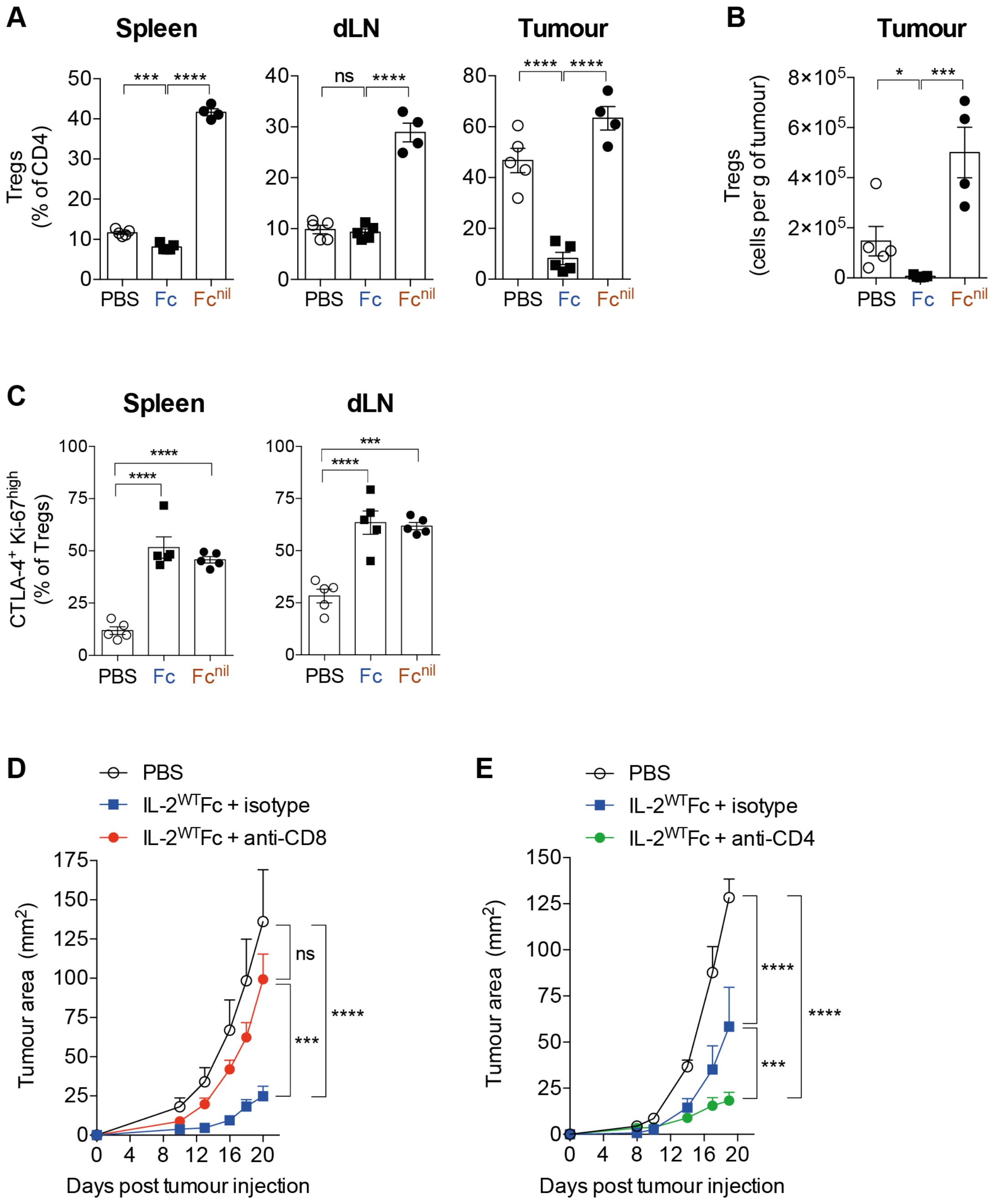

Supplementary Figure 9: IL-2^{WT}Fc treatment mediates Treg depletion at the tumour site and its efficacy is dependent on CD8^{+} T-cell activity. (A-B) Flow cytometric analysis of spleens, dLNs (inguinal) and B16F10 tumours harvested from C57BL/6 mice receiving IL-2^{WT}Fc, IL-2^{WT}Fc^{nil} or PBS control treatment (n = 4-5). Following s.c. tumour inoculation (day 0), mice received a total of five doses of IL-2-Fc on days 12-16 (5.6 μg per dose, i.p.), followed by analysis 24 h after the last dose (day 17). (A) Frequency of FoxP3^{+} cells (percentage of CD4^{+}) in the spleens, dLNs

and tumours of treated mice, showing Treg-depletive activity of IL-2^{WT}Fc relative to its Fc^{nil} counterpart. (B) Numbers of $CD4^{+}$ $FoxP3^{+}$ cells per gram of tumour. (C) Percentages of CTLA-4^{+} Ki-67^{high} Tregs in the spleens and dLNs of IL-2-Fc-treated C57BL/6 mice bearing B16F10 tumours, as determined by flow cytometry 48 h after the last IL-2-Fc dose (n = 5, see Figures 5B-I for dosing). (D-E) Efficacy of IL-2^{WT}Fc treatment after antibody-mediated depletion of total $CD8^{+}$ (D) or total $CD4^{+}$ (E) T-cells. C57BL/6 mice received anti-mouse CD8 (clone YTS 169.4), anti-mouse CD4 (clone GK1.5) or rat IgG2b isotype control (clone LTF-2) antibody on days -5, -3, -1 and +3 (100 μg per injection, i.p.). Subcutaneous inoculation of B16F10 tumours was performed on day 0 and mice received a standard course of IL-2^{WT}Fc treatment (5.6 μg per dose, i.p.) or PBS control on days 1-5 (n = 5). Monoclonal antibodies were purchased from Bio X Cell (Lebanon, NY). Data are displayed as mean ± SEM. Significant differences as determined by one-way (panels A-C) or two-way (panels D and E) ANOVA with Bonferroni post hoc test for multiple comparisons (* $P < 0.05$, ** $P < 0.01$, *** $P < 0.001$, **** $P < 0.0001$).

SUPPLEMENTARY DISCUSSION

Generation of IL-2 mutants devoid of CD25 interactions

Single mutants were expressed as Fc-fusions in HEK293 cells, purified by affinity chromatography, and ranked based on their retained binding to bivalent human CD25 (hCD25) using biolayer interferometry (Supplementary Figures 1B and 1C). This revealed that two of the substitutions, R38D and E61R, had the most pronounced effect on hCD25 binding. Surface plasmon resonance (SPR) measurements revealed that these mutations caused 20-70 fold reductions in affinity to monovalent hCD25 relative to the wild-type protein (Supplementary Figures 1D and 1E). Moreover, their combination in a double mutant (IL-2^{2X}Fc) further reduced affinity to undetectable levels (K_D >> 10 μM, Supplementary Fig. 1D). Consistent with these results, IL-2^{R38D}Fc, IL-2^{E61R}Fc and IL-2^{2X}Fc displayed nearly identical disruptions in binding to mouse CD25 (Supplementary Fig. 1G). A similar profile was observed in *in vitro* assays using the $CD25^{high}$ murine cell line CTLL-2, in which IL-2^{2X}Fc was the only variant to display complete abolition of binding and substantial reductions in proliferation and signalling (Supplementary Figures 1A and B). In contrast, introduced mutations only showed minimal reductions in activation of $CD122^{high}$ cells (Supplementary Fig. 2C) and affinity to recombinant CD122 (Supplementary Fig. 1F).

Assessment of *in vivo* activity revealed that IL-2^{2X}Fc mediated vigorous expansion of $CD122^{high}$ memory-phenotype (MP) $CD8^+$ T-cells and NK cells in the spleens of C57BL/6 mice, considerably higher than that observed after treatment with IL-2/mAb complexes, IL-2^{WT}Fc, IL-2^{R38D}Fc or IL-2^{E61R}Fc (Supplementary Fig. 3A).

This activity was also superior to that of IL-2^{F42A}Fc (Supplementary Fig. 3A), which incorporates a previously reported CD25-disrupting mutation[1] that we found was insufficient to abolish hCD25 binding (Supplementary Figures 1E and 1F). The remarkable increase in the numbers of MP CD8 (30-fold) and NK cells (16-fold) after single dose IL-2^{2X}Fc administration resulted from a combination of increased subset frequencies (Supplementary Fig. 3C), increased spleen lymphoid cellularity (Supplementary Fig. 3B), induction of a major shift in the CD8^{+} to CD4^{+} T-cell ratio (Supplementary Fig. 3D) and prolongation of serum half-life (Supplementary Fig. 3E). Although a prolonged serum half-life was not anticipated, this is likely the result of abolished interaction with CD25, since this IL-2R subunit is known to participate in IL-2 homeostasis[8]. Half-life prolongation was further validated using an *in vivo* reporter assay, in which a single dose of IL-2^{2X}Fc given 48 h before adoptive transfer of MP CD8 cells was able to drive their proliferation (Supplementary Fig. 3F).

Although the disruption of CD25 binding in the IL-2^{2X}Fc double mutant resulted in preferential expansion of MP CD8 and NK cells, this variant also induced a lower but considerable expansion of CD4^{+} CD25^{+} Tregs (5-fold, Supplementary Fig. 3A). This might be explained by the ability of IL-2^{2X}Fc to induce residual levels of proliferation and signalling in CD25^{high} cells *in vitro* (Supplementary Fig. 2B). Thus, to further improve selectivity, we generated triple mutants combining the identified R38D and E61R mutations with a range of additional substitutions. Addition of the K43D mutation tested in our initial scan (Supplementary Figures 1B and 1C) resulted in poor expression yields and low biological activity (Supplementary Fig. 4A), suggesting that a delicate structural balance needs to be maintained in order to prevent activity loss. Therefore, we generated a panel of triple mutants in order to

identify permissible substitutions at position 43 (Supplementary Fig. 4B). *In vivo* screening in C57BL/6 mice led to the identification of a triple mutant with exquisite specificity towards $CD122^{high}$ subsets, carrying the charge reversal substitution K43E (Figure 1A and Supplementary Fig. 4C). This triple mutant ($IL\text{-}2^{3X}Fc$) displayed potent activity and an increased expansion ratio of MP CD8 and NK cells over Tregs, which remained at levels comparable to PBS control mice (Supplementary Figures 4D and 4E). This remarkable selectivity resulted from further reductions in proliferation and activation of $CD25^{+}$ cells, both *in vitro* (Supplementary Figures 4H and 4I) and *in vivo* (Supplementary Figures 4E-4G).

We next compared the cellular biodistribution profiles of fluorescently labelled $IL\text{-}2^{WT}Fc$, $IL\text{-}2^{2X}Fc$ and the selected $IL\text{-}2^{3X}Fc$ in the spleens of treated mice (Supplementary Figures 5A and 5B). Importantly, association with total $CD4^{+}$ T-cells was reduced as the number of CD25-disrupting mutations increased (F Supplementary Fig. 5E). Since the majority of IL-2-Fc binding in the $CD4^{+}$ compartment is mediated by Tregs (Supplementary Fig. 5C), this suggests that $IL\text{-}2^{2X}Fc$ and $IL\text{-}2^{3X}Fc$ have a reduced ability to target this subset. Indeed, $IL\text{-}2^{WT}Fc$ bound efficiently to Tregs (~ 82% $IL\text{-}2\text{-}Fc^{+}$), which was markedly reduced for $IL\text{-}2^{2X}Fc$ (~ 8% $IL\text{-}2\text{-}Fc^{+}$) and abolished for $IL\text{-}2^{3X}Fc$ (< 0.5% $IL\text{-}2\text{-}Fc^{+}$) (Supplementary Figures 5A, 5B and 5F). Although the $IL\text{-}2^{3X}Fc$ triple mutant displayed reduced retention in the spleen (Supplementary Fig. 5C) it targeted more than 95% of NK cells in this organ, thus confirming its ability to interact with $CD122^{high}$ $CD25^{-}$ subsets (Supplementary Figures 5A and 5B).

SUPPLEMENTARY REFERENCES

1 Weir, M. P., Chaplin, M. A., Wallace, D. M., Dykes, C. W. & Hobden, A. N. Structure activity relationships of recombinant human interleukin-2. *Biochemistry (Mosc.)* **27**, 6883-6892, doi:10.1021/bi00418a034 (1988).

2 Levin, A. M. *et al.* Exploiting a natural conformational switch to engineer an interleukin-2 'superkine'. *Nature* **484**, 529-533, doi:10.1038/nature10975 (2012).

3 Duncan, A. R. & Winter, G. The binding site for C1q on IgG. (1988).

4 Wines, B. D., Powell, M. S., Parren, P. W., Barnes, N. & Hogarth, P. M. The IgG Fc contains distinct Fc receptor (FcR) binding sites: the leukocyte receptors FcγRI and FcγRIIa bind to a region in the Fc distinct from that recognized by neonatal FcR and protein A. *The Journal of Immunology* **164**, 5313-5318 (2000).

5 Edelman, G. M. *et al.* The covalent structure of an entire γG immunoglobulin molecule. *Proceedings of the National Academy of Sciences* **63**, 78-85 (1969).

6 Sondermann, P., Huber, R., Oosthuizen, V. & Jacob, U. The 3.2-Å crystal structure of the human IgG1 Fc fragment–FcγRIII complex. *Nature* **406**, 267-273 (2000).

7 Boyman, O., Kovar, M., Rubinstein, M. P., Surh, C. D. & Sprent, J. Selective stimulation of T cell subsets with antibody-cytokine immune complexes. *Science* **311**, 1924-1927, doi:10.1126/science.1122927 (2006).
